# Effect of smoking on physical and cognitive capability in later life: a multicohort study using observational and genetic approaches

**DOI:** 10.1136/bmjopen-2015-008393

**Published:** 2015-12-15

**Authors:** Teri-Louise North, Tom M Palmer, Sarah J Lewis, Rachel Cooper, Chris Power, Alison Pattie, John M Starr, Ian J Deary, Richard M Martin, Avan Aihie Sayer, Meena Kumari, Cyrus Cooper, Mika Kivimaki, Diana Kuh, Yoav Ben-Shlomo, Ian N M Day

**Affiliations:** 1 School of Social and Community Medicine, University of Bristol, Bristol, UK; 2 Division of Health Sciences, Warwick Medical School, University of Warwick, Warwick, UK; 3 Department of Mathematics and Statistics, Lancaster University, Lancaster, UK; 4 MRC Unit for Lifelong Health and Ageing at UCL, London, UK; 5 Population, Policy and Practice, UCL Institute of Child Health, University College London, London, UK; 6 Department of Psychology, University of Edinburgh, Edinburgh, UK; 7 Centre for Cognitive Ageing and Cognitive Epidemiology, University of Edinburgh, Edinburgh, UK; 8 University of Bristol/University Hospitals Bristol NHS Foundation Trust National Institute for Health Research Bristol Nutrition Biomedical Research Unit, University of Bristol, Bristol, UK; 9 MRC Lifecourse Epidemiology Unit, University of Southampton, Southampton, UK; 10 ISER, University of Essex, Essex, UK; 11 Department of Epidemiology and Public Health, UCL, London, UK; 12 National Institute for Health Research Nutrition Biomedical Research Centre, University of Southampton and University Hospital Southampton NHS Foundation Trust, Southampton, UK; 13 National Institute for Health Research Musculoskeletal Biomedical Research Unit, University of Oxford, Oxford, UK

**Keywords:** EPIDEMIOLOGY, GENETICS, GERIATRIC MEDICINE

## Abstract

**Objectives:**

The observed associations between smoking and functional measures at older ages are vulnerable to bias and confounding. Mendelian randomisation (MR) uses genotype as an instrumental variable to estimate unconfounded causal associations. We conducted a meta-analysis of the observational associations and implemented an MR approach using the smoking-related single nucleotide polymorphism rs16969968 to explore their causal nature.

**Setting:**

9 British cohorts belonging to the HALCyon collaboration.

**Participants:**

Individual participant data on N=26 692 individuals of European ancestry (N from earliest phase analysed per study) of mean ages 50–79 years were available for inclusion in observational meta-analyses of the primary outcomes.

**Primary outcomes:**

Physical capability, cognitive capability and cognitive decline. The smoking exposures were cigarettes per day, current versus ex-smoker, current versus never smoker and ever versus never smoker.

**Results:**

In observational analyses current and ever smoking were generally associated with poorer physical and cognitive capability. For example, current smokers had a general fluid cognition score which was 0.17 z-score units (95% CI −0.221 to −0.124) lower than ex-smokers in cross-sectional analyses. Current smokers had a walk speed which was 0.25 z-score units lower than never smokers (95% CI −0.338 to −0.170). An MR instrumental variable approach for current versus ex-smoker and number of cigarettes smoked per day produced CIs which neither confirmed nor refuted the observational estimates. The number of genetic associations stratified by smoking status were consistent with type I error.

**Conclusions:**

Our observational analysis supports the hypothesis that smoking is detrimental to physical and cognitive capability. Further studies are needed for a suitably powered MR approach.

**Strengths and limitations of this study**

- Using individual participant data from nine UK cohorts of ageing individuals, this study meta-analyses the associations between smoking and physical and cognitive capability in later life.
- We consider cognitive and physical capability, in addition to cognitive capability decline.
- We derive a score for general fluid cognition and include this in cross-sectional analyses.
- We use the rs16969968 single nucleotide polymorphism, which associates with nicotine dependence, in a Mendelian randomisation (MR) to explore the causality of the observational associations.
- While our study has demonstrated the feasibility of using an MR approach to understand the association of smoking with ageing outcomes, it has demonstrated that a larger sample size is required for a suitably powered analysis.

## Introduction

Epidemiological studies have been conducted to explore the associations between smoking and physical and cognitive capability in mid to later life, generally concluding that smoking is associated with worse capability outcomes.^1–8^ Physical and cognitive capability, otherwise known as the physical and intellectual tasks of daily living, are often used as markers of ageing having been consistently shown to be associated with survival and onset of disease and disability. For example, a recent meta-analysis of over 30 studies showed that poorer objective physical capability is associated with higher mortality rates.^9^ Smoking is a modifiable behaviour and understanding the extent to which it influences biological ageing is crucial given the burden of morbidity in today's ageing populations.

The associations between smoking and markers of ageing are likely to be confounded (or mediated) by factors such as socioeconomic position (SEP), body mass index, health status and prior IQ. Although studies have adjusted for these factors, residual confounding and bias may have affected the interpretation of results. Mendelian randomisation (MR) uses genotype as an instrumental variable (IV) to estimate the causal effect of an exposure on an outcome free of confounding and reverse causation bias.^10^ ^11^ If genotype is associated with the exposure under consideration and is not associated with the confounders of the observational association, nor directly with the outcome of interest, it may be used to conduct an IV analysis to generate a causal estimate of the observational association. This can be implemented, for example, using a two-stage approach where the predicted exposure based on genotype is used to measure the association with the outcome. Associations of genotype with the outcome in different strata of the exposure can also contest or support the causality of the observational association. If a genetic association is observed in exposed individuals and not in unexposed individuals, this supports a causal observational association. The minor allele of the rs16969968 single nucleotide polymorphism (SNP) in the *CHRNA5* gene has been consistently associated with increased nicotine dependence,^12^ therefore providing a potential instrument for MR analyses of the effects of smoking.

Harnessing the availability of data across cohorts belonging to the HALCyon collaboration,^13^ we have conducted a meta-analysis of smoking and physical and cognitive capability using individual participant data (IPD) from middle-aged to older individuals in the UK. Given the known associations between rs16969968 and nicotine dependence, we explored the associations between this SNP and our ageing outcomes in different smoking classes to supplement the observational associations. We considered that if associations are observed in current or ever smokers but not in never smokers, this provides evidence to support causality. We implemented instrumental variable regression to generate IV estimates for the true causal associations of smoking with continuous physical and cognitive capability measures. Our aim was to examine whether the observational associations are consistent with estimates obtained using an MR approach.

## Method

IPD was meta-analysed across nine cohorts belonging to the HALCyon collaboration. These included the Boyd Orr Cohort (BO), the Caerphilly Prospective Study (CaPS), the English Longitudinal Study of Ageing (ELSA), the Hertfordshire Ageing Study (HAS), the Hertfordshire Cohort Study (HCS), the Lothian Birth Cohort 1921 (LBC1921), the National Child Development Study (NCDS), the MRC National Survey of Health and Development (NSHD) and the Whitehall II Study (WHII). Further information about the cohorts is provided in previous HALCyon publications.^14^

### Outcome measures

Physical capability was assessed using the objective measures grip strength, chair rise speed, inability to balance on one leg for 5 s with eyes open, walking speed and timed get up and go (TUG) speed. Cognitive capability measures included tests of crystallised intelligence (National Adult Reading Test (NART) and Mill Hill) and fluid intelligence (Alice-Heim 4-I test (AH4), semantic fluency, phonemic fluency, search speed, word recall, four choice reaction time (FCRT), logical memory and Raven's Progressive Matrices). Crystallised intelligence measures knowledge accumulated across the life course like vocabulary and captures premorbid IQ, while fluid intelligence measures problem-solving skills.^15^ Cognitive decline was calculated by taking the percentage change in continuous cognitive measure between the baseline wave and the last available wave. This was then converted to the percentage change per year using the age difference between waves. Individuals were categorised into a binary variable of the top 25% of decliners versus others in each cohort. This approach is similar to previous studies.^16^ ^17^ We used factor analysis to derive a general fluid (Gf) cognitive ability score and included this in the cross-sectional analyses.

### Smoking behaviour

Participants were classified at the time of baseline capability assessment as current, ex or never smokers. A ‘smoker’ was defined as an individual who smoked pipes, cigars, manufactured cigarettes or hand-rolled cigarettes, if this information was available. For current smokers, we additionally analysed number of cigarettes smoked per day (CPD). Where possible this was restricted to manufactured cigarettes to maintain consistency in tobacco quantities. Individuals who were occasional smokers (smokes less than 1 CPD or does not smoke daily but does smoke occasionally) were re-coded as smoking 0 CPD. In Boyd Orr, we estimated CPD by taking the median of intervals of cigarettes smoked per day.

### Genetic analyses

We genotyped rs16969968 across cohorts where this was not previously available. Rs1051730 was substituted into analyses where available and when rs16969968 was unavailable (see online supplementary table S1). These two SNPs are highly correlated and thus interchangeable.^12^

### Covariates

We selected a range of covariates a priori. These were sex, age (continuous), socioeconomic position obtained from the earliest wave of outcome assessment (the Registrar-General's Social Class, RGSC), body mass index (BMI), height and disease status (history of diabetes, stroke or heart disease: see online supplementary table S2). BMI, height and disease status were derived using data from the same wave as each outcome measure if available unless the outcome was a decline measure in which case the covariates were taken from the baseline wave. Age, sex and SEP were included as potential confounders of the observational associations of smoking and physical and cognitive capability. Disease history was included to examine the extent of mediation of smoking effects via diabetes, stroke and heart disease. BMI may confound and mediate the association of smoking and physical capability, while height is strongly correlated with physical capability outcomes.

Further information on the genotyping and the derivation and harmonisation of the exposures, covariates and outcomes is provided in online supplementary material.

### Statistical analysis

All analyses were conducted using Stata 13.1.^18^ Known ethnic outliers were excluded from the analysis (self-reported non-European ancestry or previously detected from genome-wide data), as were related individuals. For harmonisation continuous outcome measures were standardised within cohorts using all data available. Logistic regression was implemented for analyses of binary outcomes and linear regression for continuous outcomes.

Four choice reaction time was inverse transformed and search speed was natural log transformed to improve normality. A score for Gf was derived in several cohorts using the -factor- command in Stata^18^ using the pcf option and imposing one factor, to supplement cross-sectional analyses.

Observational analyses assessed the associations between CPD or smoking status (current vs ex, current vs never or ever vs never smoker) and each of the physical and cognitive capability measures. Three models were run for physical capability adjusted for (1) age, SEP and sex (2) age, SEP, sex and disease status (3) age, SEP, sex, disease status, height and BMI. Models (1) and (2) were run for the cognitive outcomes.

The rs16969968 genotype was coded additively as 0, 1 or 2 minor alleles. The associations between genotype and smoking behaviour were calculated using linear or logistic regression. The associations between genotype and physical and cognitive capability were calculated in current, never and ever smokers to test for both pleiotropy and whether an association was observed in current and ever but not never smokers. All genetic associations were adjusted for age and sex.

For the smoking exposures which correlated with genotype (cigarettes per day and current vs ex smoking) and for the continuous outcomes which associated with these smoking exposures, we performed IV estimation using the two-stage least squares (2SLS) estimator and compared the observational estimates with the IV estimates. The IV assumptions, that can be checked using observational data, were checked by testing the unadjusted associations between genotype and the covariates.

All associations were analysed within cohorts and the effect estimates meta-analysed using a random effects two-stage approach.^19^ ^20^ The observational analyses included all individuals with data available on the exposures and outcomes of interest. Secondary models were restricted to individuals who had relevant covariates available. The genetic and IV analyses on the associations between genotype and cognitive and physical capability were conducted on a subset of the observational sample with genotype data. The associations between genotype and smoking behaviour or covariates were examined in all individuals with data available.

## Results

The observational and genetic samples are characterised in table 1 and online supplementary tables S3–5. The mean age in the observational analysis was 50–79 years and the majority of studies had similar numbers of men and women. The total sample size taking the earliest wave of outcome assessment was 26 692. As shown in table 2, each of the physical capability outcomes was associated with at least one comparison of smoking status. In particular, current compared to never smoker status resulted in a decrease in walking or TUG speed of between 0.23 and 0.29 z-scores (p<0.0001). Across outcomes and models, the effect estimates generally suggest that being a current or an ever smoker was associated with worse physical capability compared to never smokers. Adjustment for BMI and height often resulted in an increased magnitude of effect on physical capability for the smoking exposures. Heterogeneity statistics are provided in the Supplement.

**Table 1:** Age and sex by cohort study

Cohort	Mean age in years (SE)	Percentage female	Total sample size, n
BO	69.64 (0.25)	55.56	279
CaPS	61.77 (0.10)	0	1831
ELSA	66.01 (0.13)	54.56	5425
HAS	67.39 (0.09)	35.85	636
HCS	66.13 (0.05)	47.84	2803
LBC	79.06 (0.02)	57.38	542
NCDS	50 (NA)	50.63	7652
NSHD	53.45 (0.00)	50.9	2949
WHII	55.39 (0.09)	26.56	4575
TOTAL			26 692

Numbers based on all individuals with age, sex, smoking status, socioeconomic position (SEP) and at least one outcome measure at the earliest phase analysed. Numbers based on grip strength analysis for HCS.

**Table 2:** Observational estimates for the associations between smoking and physical capabilities

Outcome	Model†	Cigarette per day‡	Current vs ex-smoker	Current vs never smoker	Ever vs never smoker
*Regression coefficient (95% CI)*					
Grip strength	M1	0.000 (−0.003 to 0.003)	−0.049** (−0.085 to −0.014)	−0.017 (−0.077 to 0.043)	−0.002 (−0.045 to 0.041)
	M2	0.001 (−0.003 to 0.004)	−0.054** (−0.088 to −0.019)	−0.012 (−0.069 to 0.045)	0.006 (−0.034 to 0.047)
	M3	0.001 (−0.003 to 0.004)	−0.026 (−0.060 to 0.008)	−0.006 (−0.051 to 0.040)	−0.001 (−0.040 to 0.038)
Chair rise speed	M1	−0.004 (−0.010 to 0.001)	−0.111** (−0.190 to −0.032)	−0.150*** (−0.233 to −0.067)	−0.061** (−0.102 to −0.020)
	M2	−0.004 (−0.010 to 0.002)	−0.115** (−0.188 to −0.043)	−0.152*** (−0.242 to −0.062)	−0.059* (−0.108 to −0.010)
	M3	−0.002 (−0.008 to 0.004)	−0.152**** (−0.224 to −0.079)	−0.188*** (−0.285 to −0.091)	−0.052* (−0.095 to −0.009)
Walk speed	M1	−0.006 (−0.016 to 0.005)	−0.129* (−0.242 to −0.016)	−0.254**** (−0.338 to −0.170)	−0.136**** (−0.171 to −0.102)
	M2	−0.006 (−0.015 to 0.002)	−0.142* (−0.250 to −0.033)	−0.247**** (−0.325 to −0.168)	−0.124**** (−0.159 to −0.089)
	M3	−0.001 (−0.013 to 0.010)	−0.185** (−0.304 to −0.066)	−0.266**** (−0.373 to −0.159)	−0.110**** (−0.144 to −0.075)
TUG speed	M1	0.000 (−0.010 to 0.011)	−0.075 (−0.161 to 0.011)	−0.233**** (−0.323 to −0.144)	−0.138** (−0.234 to −0.042)
	M2	0.001 (−0.009 to 0.012)	−0.102* (−0.186 to −0.017)	−0.236**** (−0.331 to −0.142)	−0.123* (−0.217 to −0.029)
	M3	0.007 (−0.007 to 0.021)	−0.166**** (−0.249 to −0.082)	−0.290**** (−0.383 to −0.197)	−0.132*** (−0.205 to −0.060)
*OR (95% CI)*					
Inability to balance on one leg for 5 s	M1	1.015 (0.994 to 1.036)	1.125 (0.937 to 1.351)	1.210 (0.948 to 1.543)	1.092 (0.957 to 1.246)
	M2	1.013 (0.992 to 1.035)	1.155 (0.958 to 1.392)	1.232* (1.000 to 1.516)	1.064 (0.930 to 1.217)
	M3	1.007 (0.985 to 1.029)	1.361** (1.120 to 1.655)	1.415** (1.106 to 1.811)	1.074 (0.934 to 1.236)

*p<0.05, **p<0.01, ***p<0.001, ****p<0.0001. †Models: (M1) age, sex and SEP adjusted, (M2) M1 + disease adjusted, (M3) M2 + height, BMI adjusted. ‡Association is for 1 CPD for comparison with genotypic analysis.

BMI, body mass index; CPD, cigarettes smoked per day; SEP, socioeconomic position; TUG, timed get up and go.

Current compared to ex or never smokers performed more poorly across all cross-sectional cognitive outcomes tested for both models with the exception of phonemic fluency and the single cohort analyses (table 3, see online supplementary table S6). An extra cigarette per day was associated with a slower search speed (p<0.01 in model 2) and there was a trend towards poorer word recall ability.

**Table 3:** Observational estimates for the associations between smoking and cognitive capabilities

		Regression coefficient (95% CI)			
Outcome	Model†	Cigarette per day	Current vs ex-smoker	Current vs never smoker	Ever vs never smoker
*Crystallised measures*					
Mill Hill	M1	−0.003 (−0.012 to 0.005)	−0.140*** (−0.216 to −0.063)	−0.192**** (−0.266 to −0.117)	−0.092*** (−0.142 to −0.043)
	M2	−0.004 (−0.013 to 0.005)	−0.146*** (−0.223 to −0.070)	−0.194**** (−0.269 to −0.119)	−0.090*** (−0.140 to −0.040)
NART	M1	−0.004 (−0.010 to 0.001)	−0.174**** (−0.261 to −0.088)	−0.159** (−0.262 to −0.056)	−0.041 (−0.097 to 0.016)
	M2	−0.004 (−0.010 to 0.002)	−0.194**** (−0.265 to −0.123)	−0.142* (−0.260 to −0.023)	−0.028 (−0.086 to 0.030)
*Fluid measures*					
Gf	M1	−0.002 (−0.011 to 0.007)	−0.173**** (−0.221 to −0.124)	−0.147**** (−0.205 to −0.088)	−0.036* (−0.067 to −0.006)
	M2	−0.002 (−0.011 to 0.007)	−0.178**** (−0.228 to −0.129)	−0.137**** (−0.199 to −0.074)	−0.029 (−0.059 to 0.002)
AH4	M1	0.000 (−0.008 to 0.009)	−0.139**** (−0.195 to −0.082)	−0.135**** (−0.196 to −0.073)	−0.047* (−0.090 to −0.003)
	M2	0.000 (−0.009 to 0.009)	−0.148**** (−0.206 to −0.090)	−0.129**** (−0.192 to −0.065)	−0.040 (−0.084 to 0.004)
Semantic fluency	M1	−0.002 (−0.008 to 0.003)	−0.139**** (−0.175 to −0.104)	−0.105*** (−0.162 to −0.047)	−0.019 (−0.059 to 0.021)
	M2	−0.002 (−0.007 to 0.003)	−0.136**** (−0.172 to −0.100)	−0.095** (−0.155 to −0.035)	−0.012 (−0.053 to 0.029)
Phonemic fluency	M1	−0.005 (−0.015 to 0.006)	−0.028 (−0.378 to 0.322)	0.041 (−0.289 to 0.372)	0.031 (−0.022 to 0.084)
	M2	−0.005 (−0.015 to 0.005)	−0.063 (−0.355 to 0.228)	0.056 (−0.295 to 0.407)	0.037 (−0.016 to 0.091)
Search speed‡	M1	−0.005* (−0.009 to −0.001)	−0.122*** (−0.188 to −0.057)	−0.148** (−0.239 to −0.057)	−0.059** (−0.099 to −0.019)
	M2	−0.006** (−0.010 to −0.002)	−0.122*** (−0.192 to −0.052)	−0.142** (−0.232 to −0.051)	−0.054** (−0.091 to −0.016)
Word recall	M1	−0.005 (−0.010 to 0.000)	−0.144*** (−0.222 to −0.067)	−0.138**** (−0.192 to −0.083)	−0.044*** (−0.071 to −0.018)
	M2	−0.005 (−0.010 to 0.000)	−0.143*** (−0.222 to −0.064)	−0.134**** (−0.191 to −0.078)	−0.042** (−0.069 to −0.016)

*p<0.05, **p<0.01, ***p<0.001, ****p<0.0001.

†Models: (M1) age, sex and SEP adjusted, (M2) M1 + disease adjusted.

‡Natural log transformed.

AH4, Alice-Heim 4-I test; GF, general fluid; NART, National Adult Reading Test; SEP, socioeconomic position.

There were fewer associations apparent between smoking behaviour and cognitive decline (see online supplementary tables S7 and 8). Notably, current compared to ex-smokers were more likely to be in the quartile of greatest decliners in AH4 score, word recall ability, search speed and FCRT. Current smokers experienced worse decline than never smokers in word recall and FCRT, while ever smokers declined faster on the Raven's Progressive Matrices than never smokers. Adjustment for disease status had a small influence on the effect sizes for cognitive outcomes in general.

### Instrumental variable analysis

**Figure 1:**
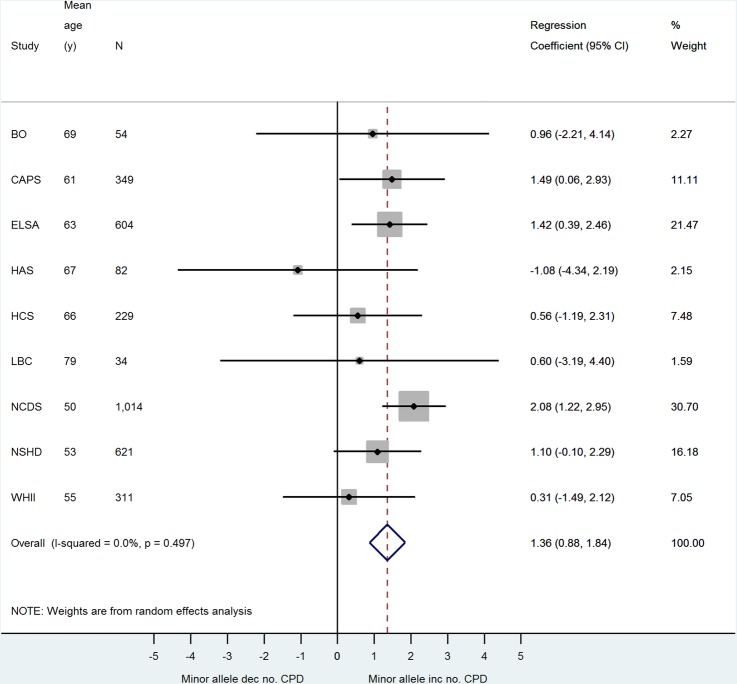
Meta-analysis of minor allele—cigarettes smoked per day association.

**Figure 2:**
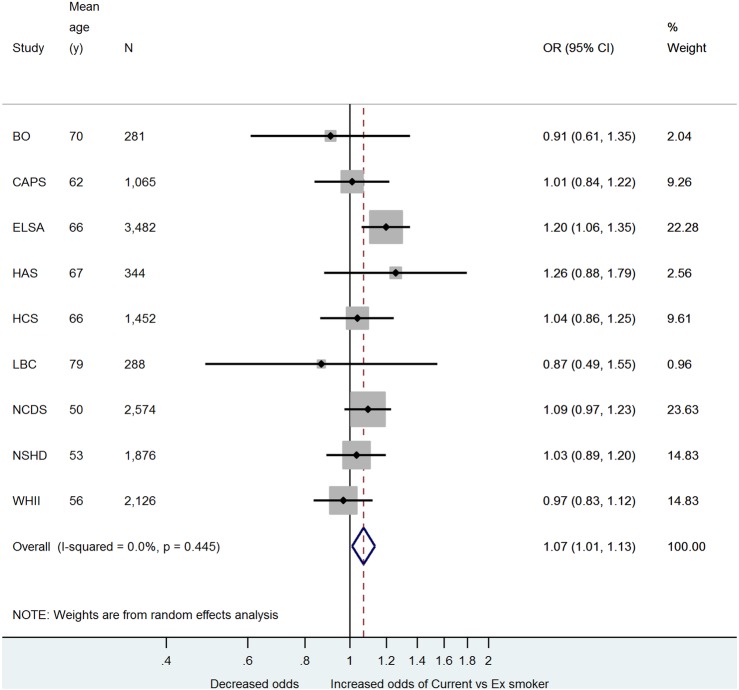
Meta-analysis of minor allele—current versus ex-smoker association.

The association between rs16969968 and smoking behaviour is summarised in figures 1 and 2 and online supplementary figures 1 and 2. Each minor allele predicts approximately 1 extra cigarette smoked per day (figure 1). The F statistic^21^ for the strength of the association between each minor allele and CPD, obtained from the partial R^2^ value from the first stage of the age and sex-adjusted 2SLS regression of CPD and log-transformed search speed, was 3.51 in NSHD, 7.18 in ELSA and 13.64 in NCDS. We also observed an association between each extra minor allele and an increased odds of being a current compared with an ex-smoker (figure 2, p=0.02). There was some evidence of an association between the SNP and being an ever versus a never smoker (see online supplementary figure 1, decreased odds of ever smoker p=0.05), but no evidence for an association with being a current versus a never smoker (see online supplementary figure 2).

There was no evidence for an association between rs16969968 and the covariates, although the age distribution was non-normal (see online supplementary table S9). We tested the association between rs16969968 and each outcome in current, never and in ever smokers (table 4, see online supplementary table S10). We observed an association between the smoking susceptibility allele and increased odds of being in the top 25% of decliners for FCRT in current smokers (see online supplementary table S10). In never smokers, we observed an association between the same allele and poorer search speed.

**Table 4:** Associations between rs16969968 and outcomes stratified by smoking status

Outcome category	Outcome	Current smokers	Never smokers	Ever smokers
*Regression coefficient (95% CI)*				
Physical capability	Grip strength	0.008 (−0.044 to 0.061)	0.016 (−0.030 to 0.062)	−0.015 (−0.037 to 0.008)
	Chair rise speed	0.003 (−0.123 to 0.130)	0.000 (−0.078 to 0.079)	−0.002 (−0.048 to 0.044)
	Walk speed	0.049 (−0.122 to 0.220)	0.026 (−0.015 to 0.068)	0.002 (−0.033 to 0.038)
	TUG speed	−0.043 (−0.154 to 0.067)	−0.015 (−0.083 to 0.054)	−0.024 (−0.077 to 0.029)
Cognitive capability	Mill Hill	0.062 (−0.058 to 0.182)	−0.031 (−0.096 to 0.034)	−0.042 (−0.214 to 0.129)
	NART	0.020 (−0.075 to 0.116)	−0.009 (−0.098 to 0.080)	−0.004 (−0.058 to 0.051)
	Gf	0.048 (−0.011 to 0.108)	−0.035 (−0.074 to 0.004)	0.016 (−0.015 to 0.047)
	AH4	0.024 (−0.069 to 0.118)	−0.040 (−0.102 to 0.021)	0.013 (−0.038 to 0.064)
	Semantic fluency	0.028 (−0.038 to 0.094)	−0.013 (−0.048 to 0.022)	0.014 (−0.014 to 0.042)
	Phonemic fluency	−0.033 (−0.160 to 0.094)	−0.018 (−0.085 to 0.048)	−0.126 (−0.398 to 0.145)
	Search speed†	0.024 (−0.045 to 0.093)	−0.060* (−0.116 to −0.003)	0.005 (−0.028 to 0.038)
	Word recall	0.045 (−0.009 to 0.099)	−0.018 (−0.058 to 0.023)	0.015 (−0.014 to 0.044)
*OR (95% CI)*				
Physical capability	Inability to balance on one leg for 5 s	1.010 (0.732 to 1.394)	1.052 (0.896 to 1.236)	0.921 (0.817 to 1.038)
Cognitive capability decline	Mill Hill	0.535 (0.063 to 4.559)	0.943 (0.798 to 1.114)	0.935 (0.802 to 1.089)
	NART	1.016 (0.678 to 1.522)‡	0.878 (0.559 to 1.379)	1.085 (0.848 to 1.388)
	AH4	1.007 (0.786 to 1.289)	1.127 (0.955 to 1.329)	0.925 (0.807 to 1.060)
	Semantic fluency	0.975 (0.748 to 1.271)	1.243 (0.926 to 1.670)	0.984 (0.891 to 1.086)
	Phonemic fluency	1.003 (0.735 to 1.369)§	1.097 (0.807 to 1.492)	1.163 (0.565 to 2.393)
	Word recall	0.999 (0.832 to 1.201)	1.024 (0.913 to 1.149)	0.978 (0.890 to 1.074)

Models adjusted for age and sex.

*p<0.05, **p<0.01, ***p<0.001, ****p<0.0001.

†Natural log transformed.

‡Analysis in CAPS only.

§Analysis in WHII only.

AH4, Alice-Heim 4-I test; Gf, general fluid; NART, National Adult Reading Test; TUG, timed get up and go.

Given the strong association between rs16969968 and CPD, and the weaker association with this variant and current versus ex-smoker status, we progressed all observational associations for these exposures (arbitrary threshold of p<0.05) to an IV analysis. The results are described in table 5 (and see online supplementary table S1^1^). The CIs from the observational estimates fall within the CIs from the IV estimates. The IV results, however, are not informative about the causality of the observational associations because the IV CIs are wide.

**Table 5:** Comparison of observational with instrumental variable estimates

Observational association of interest		Observational estimate, β_O_† (95% CI)	IV estimate, β_IV_ (95% CI)
Smoking behaviour	Outcome		
Cigarettes per day	Search speed‡	−0.005* (−0.009 to −0.001)	0.017 (−0.041 to 0.075)
Current vs ex-smoker	Grip strength	−0.049** (−0.085 to −0.014)	−0.417 (−1.344 to 0.510)
	Walk speed	−0.129* (−0.242 to −0.016)	−0.411 (−2.008 to 1.186)
	TUG speed^M3^	−0.166**** (−0.249 to −0.082)	−0.941 (−3.937 to 2.055)
	Chair rise speed	−0.111** (−0.190 to −0.032)	−0.039 (−1.398 to 1.319)
	NART	−0.174**** (−0.261 to −0.088)	−1.236 (−8.711 to 6.238)
	Mill Hill	−0.140*** (−0.216 to −0.063)	−2.785 (−7.107 to 1.537)
	Gf	−0.173**** (−0.221 to −0.124)	0.029 (−1.394 to 1.453)
	Semantic fluency	−0.139**** (−0.175 to −0.104)	−0.154 (−1.571 to 1.263)
	AH4	−0.139**** (−0.195 to −0.082)	−1.575 (−4.707 to 1.556)
	Word recall	−0.144*** (−0.222 to −0.067)	0.247 (−1.045 to 1.538)
	Search speed‡	−0.122*** (−0.188 to −0.057)	0.312 (−1.121 to 1.744)

As explained in the methods, sample used to calculate observed and IV estimates differs according to the availability of variables.

*p<0.05, **p<0.01, ***p<0.001, ****p<0.0001.

†Observational estimates are model M1 unless stated otherwise.

‡Natural log transformed.

AH4, Alice-Heim 4-I test; Gf, general fluid; IV, instrumental variable; NART, National Adult Reading Test; TUG, timed get up and go.

## Discussion

This study has confirmed that smoking, whether it be current smoking or ever smoking, is associated with poorer grip strength, chair rise speed, TUG/walk speed and balance ability in a combined analysis of UK ageing cohorts. In addition, smoking was associated with poorer cognition across eight different cognitive tests and five measures of cognitive decline. The large sample size that HALCyon affords reveals some novel associations that, to our knowledge, have not been reported before. Notably, an association between cigarettes smoked per day and poorer search speed in current smokers. In addition, the high precision of the observational effect-estimates from this analysis are reflected in the narrow CIs. However, some of the observational association is due to residual confounding, as demonstrated by the association observed between smoking and measures of crystallised cognition which should be fairly robust to adverse environmental factors acting from young adulthood and later.

This study has analysed data across a wide range of cohorts from different geographical locations and with different age ranges. Many of the results should thus be generalisable to British individuals of European ancestry of middle to older ages. As with any cohort study, however, there may have been a healthy survivor effect and particularly the analysis of ever smokers may be applicable only to healthy smokers. All cohorts, however, demonstrated genotype frequencies within Hardy-Weinberg Equilibrium (HWE).^22^ The HWE p value for all cohorts individually was >0.3 with the exception of LBC1921 (p=0.08), while the collective p value for all cohorts combined was 0.73 (see online supplementary table S3). If the analysis is indeed confined to healthy smokers, then the negative effect of smoking on physical and cognitive capability could be biased downwards here.

Adjustment for height and BMI in the physical capability observational models often resulted in an increased magnitude of effect of smoking status on outcome. BMI could both confound and mediate the association of smoking with physical capability, so the causal relationships are likely to be complex. BMI, in addition to disease status, could also be a collider, whereby covariate adjustment induces a false association between exposure and outcome. Such issues are the primary motivation for conducting an MR. Adjustment for history of disease did not substantially alter the effect estimates in general. While this could suggest that the diseases considered are not on the causal pathway between smoking and outcome, the lack of attenuation could be because these variables have not adequately captured disease history or because they were derived using history of ever having these conditions and thus do not capture smoking-induced incident disease.

The genetic analyses detected an association between rs16969968 and poorer search speed in never smokers. We caution that this could be a false positive due to multiple testing. Of the seven cross-sectional cognitive outcomes considered in table 4 and excluding the current smokers, there were 14 independent tests. The Bonferroni corrected p value is 0.004. However, the result is in support of a hypothesis described by Winterer *et al*^23^ that suggested the mediation of the effect of the rs16969968 risk allele on greater nicotine dependence to be via poorer cognition. If the cognitive outcomes under consideration are on the causal pathway between the SNP and nicotine dependence, this violates the MR assumptions. In general, however, we conclude that the rs16969968 variant does not exert a direct effect on the outcomes that is large enough to be detectable in our sample, owing to the lack of an association in never smokers. The trend towards improved word recall ability per minor allele in current smokers complements a study on elderly Taiwanese individuals which found a protective effect of smoking on cognitive function.^24^

The association in a single cohort (CaPS) between current versus ex-smoker status and greater odds of decline in FCRT score complements the positive association of the minor allele of rs16969968 with greater odds of FCRT decline in current smokers but not never smokers. If this association is not spurious then it supports the causality of the observational association between continuing smoking and a decline in reaction ability over time.

The IV CIs did not refute the observational estimates. Our approach is a suitable framework for future studies with larger sample sizes. Taking the predictive utility of rs16969968 for CPD as an example, each additional allele predicts one extra cigarette smoked each day and accounts for approximately 1% of the variance in CPD among current smokers. Using mRnd,^25^ an online sample size calculation tool for MR, and taking a mean CPD of 14 and a variance in CPD of 81 (as per ELSA current smokers), genetic association testing in current smokers would require 961 individuals to detect an effect of 1 extra CPD on outcome of 0.1 z-score units. This sample size, which assumes 80% power and a 5% type I error rate, was achieved for several of the outcomes in HALCyon (see online supplementary table S5). However, the observed point estimate for smoking 1 extra CPD on log-transformed search speed was −0.005 z-score units, which would require a sample size of 386 815 current smokers in a 2SLS IV analysis. This demonstrates that an MR approach in HALCyon with CPD as the exposure is underpowered to detect effect sizes comparable to the observational associations, but is powered to detect moderate effect sizes. The lack of associations observed in this MR analysis suggest that the true causal effect sizes are unlikely to be moderate and are more likely to be of small magnitude. A sample size of nearly 400 000 current smokers could only be achieved via meta-analysis of consortia and inclusion of large studies such as the UK BIOBANK study (http://www.ukbiobank.ac.uk/).

While the association between the minor allele of rs16969968 and smoking an extra cigarette per day reported here is in agreement with the literature,^12^ our finding of a decreased odds of being an ever smoker with each extra minor allele is likely to be spurious. Lips *et al*^26^ found no association of smoking initiation with this SNP while Sherva *et al*^27^ found an association between the minor allele and increased odds of being a current versus a never smoker. In our analysis, the SNP appears to be associated with a decreased odds of initiating smoking but also with a decreased odds of quitting once smoking is initiated. Although there has been some evidence of an association between this variant and quitting ability in previous studies even after adjustment for smoking quantity,^28^ ^29^ this latter association has not been consistently replicated.^26^ A recent MR study which included several of the HALCyon cohorts reported a similar per allele OR^30^ for current versus ex-smoker but no association for ever versus never. As discussed above, the lack of a genetic association with current versus never smoker status that we report here adds to a body of literature reporting conflicting associations of this SNP with smoking initiation.^12^ ^27^

The F statistics extracted from the 2SLS IV analysis of CPD and natural log-transformed search speed suggest that rs16969968 could suffer from weak instrument bias. It has been suggested^21^ that pooling the data from the individual studies and conducting an IPD MR can reduce this bias. A suitably powered IV analysis of CPD and search speed could explore these approaches further. However, it has also been noted^30^ ^31^ that IV estimates generated using CPD as the exposure variable and rs16969968 as the genetic instrument will be biased because this SNP predicts other measures of tobacco exposure independently of CPD, thus violating the statistical assumptions of MR. In light of this, future MR studies of CPD and physical and cognitive capability should focus on examination of the genetic associations in current and never smokers, rather than on generating precise IV estimates of the true observational association. Such an approach, however, may also be weakened if ever smokers incorrectly report themselves to be never smokers or current smokers do not report the true levels of cigarettes smoked per day. Objective measures of tobacco exposure like cotinine levels avoid some of the problems of inaccurate self-reporting and it has been shown that rs16969968 is a strong predictor of cotinine independent of CPD.^32^ This biomarker, however, was not available across the HALCyon studies for meta-analysis. As recently highlighted,^30^ a further limitation of MR is that collider bias can occur when we stratify the genetic associations by smoking status because rs16969968 is associated with smoking status. Given the few genetic associations observed in table 4 and online supplementary table S10, which are consistent with type I error, collider bias is unlikely to have affected this analysis.

Our study could be extended in several other respects. The observational analyses could consider change in smoking behaviour over time^3^ ^33^ and, as data becomes available, decline in physical capability. Further research using a longitudinal approach with repeat data is needed in the future. In addition, further covariates could be incorporated into the observational models. The association of smoking with physical and cognitive capability is likely to be confounded by other factors such as alcohol intake, IQ and stress.

Previous studies of smaller sample size than ours have been successful at implementing an IV approach using this SNP.^34^ The success of using MR to infer causality depends on the predictive utility of the variant, in addition to the effect that smoking actually has on the outcome of interest which is less clearly understood. We have conducted an IPD meta-analysis of smoking and physical and cognitive capability in ageing UK cohorts. This is also the first study to date to use the *CHRNA5* rs16969968 variant to explore the causality of the relation between smoking and physical and cognitive capability. Although our results show that a larger sample size is required, this approach has demonstrated that MR analyses could be ‘instrumental’ in resolving the smoking-ageing question.
